# A Discussion on the Application of Terminology for Urban Soil Sealing Mitigation Practices

**DOI:** 10.3390/ijerph19148713

**Published:** 2022-07-18

**Authors:** María I. Rodríguez-Rojas, Alejandro L. Grindlay Moreno

**Affiliations:** Department of Urban and Regional Planning, Higher School of Civil Engineering, University of Granada, 18071 Granada, Spain; grindlay@ugr.es

**Keywords:** soil sealing, sustainable drainage systems, low impact developments, water sensitive urban design, nature-based solutions, best management practices, green infrastructure, environmental benefits

## Abstract

Soil sealing is one of the most serious environmental problems today regarding its impact on cities. This article presents an analysis of the different urban practices currently used to mitigate the effects of soil sealing in urban areas. The main typologies, characteristics, differences, similarities and objectives have been considered. The practices analyzed were SuDS (Sustainable Drainage Systems), LIDs (Low Impact Developments), BMPs (Best Management Practices), WSUD (Water Sensitive Urban Design), GI (Green Infrastructure), and NbS (Nature-based Solutions). To understand the impact of these terms, an analysis of their presence in the scientific literature over the last 10 years is carried out. The results indicate that the trend in the use of these terms is increasing, with the number of articles having doubled in the last 10 years. This indicates the importance that the problem of soil sealing has acquired in the world, and the relevant environmental benefits of addressing it.

## 1. Introduction

Historically the impermeabilization of urban soils was a public health measure; however, the environmental problems that have resulted as a consequence of this have meant that soil sealing has become a vital issue today. The rapid growth of urban areas and the need to facilitate road traffic has led to a process of urbanization based on waterproofing [1]. This has led to 67% of the 1000 km^2^ of land area that is urbanized per year in Europe [2] being non-permeable [3]. This phenomenon is causing enormous environmental problems in cities [4,5]; the “heat island” effect creating an increase in temperatures [6]; the saturation of wastewater treatment systems, causing the contamination of receiving waters [7,8]; and the overwhelming of the sewerage system, producing more frequent and intense flooding [9]. Sewage systems were not designed for this phenomenon, so these problems will intensify due to the effects of climate change, which predict increasingly intense rainfall [10,11].

In many cities numerous measures have been taken over the last 20 years to reduce the effects of soil sealing processes in urban areas [12,13] by mimicking hydrological conditions prior to urban development. These measures are inspired by natural processes [14,15,16], improved soil and water conservation practices, and the green economy [17,18,19]. They are mainly designed to store, infiltrate, and promote evapotranspiration as far as possible to reduce soil temperature and avoid saturation of sewage networks and thus flooding [20,21]. Therefore, these urban practices have significant environmental benefits contributing to the reduction of flooding and water pollution [7], increasing water resources [3], generating a pleasant environment, facilitating carbon sequestration, lowering temperatures in urban areas, and improving human health and well-being [22,23].

All these proven benefits have promoted in the incorporation of these measures in the environmental agendas of many cities around the world [15,16,24,25,26,27]. The integration of these practices into urban planning has modified the approach to land use and land cover, improving water supply, water quality control, soil protection, and hydrometeorological risk mitigation [25,28,29,30]. For example, less runoff is generated in urban areas and the annual water yield tends to be better than that of impermeable soils [31,32,33], which is associated with higher evapotranspiration and lower temperatures in urban areas [34].

These new practices are increasingly being applied in cities around the world under different terminologies. An example of the importance of the application of these practices is the study by Fletcher et al. [35], which analyzes the use of these terms up to 2012. The findings of their study show that those most commonly used at the end of the study period were LIDs, GI, BMPs, WSUD, and Source Control. Thus, these results have been taken as a starting point for the realization of our study, considering for terminology analysis those names that were most used in 2012. The term “Source Control” has been replaced by “Sustainable Drainage Systems”, since the latter has surpassed the former in importance in recent years, especially in Europe. On the other hand, the term “Nature-Based Solutions” has been added, which was not considered in Fletcher’s study because it is a more recently-used term. Thus, the terms that are analyzed in this article are LIDs, GI, BMPs, WSUD, SuDS, and NbS. Regarding the study period, the years from 2012 to 2021 were covered, in order to present new results in relation to the evolution of the use of these urban practices in the scientific literature over the last 10 years. The geographical scope of the study is international since, as will be explained below, international publications in the JCR database related to urban practices for soil sealing mitigation were analyzed.

## 2. Urban Practices to Mitigate Soil Sealing: Typologies, Characteristics and Objectives

The environmental consequences of soil sealing have led to the development of a multitude of measures to mitigate the effects of this phenomenon in urban areas in recent years [35]. This is illustrated by the publication “Towards an EU Research and Innovation Policy Agenda for Nature-Based Solutions & Re-naturing Cities” [14], which is one of the most important at a European and international level in relation to urban practices for soil sealing mitigation. This publication describes the basic principles of sustainable urban practices that help to reduce the effects of soil sealing. These practices, each with their own particularities, have been referred to using different terms, but of all of them their main objective is to mitigate the phenomenon of soil sealing. This group of terms is not static [35] as they constantly respond to the evolution of technologies and the incorporation of other fields to the practice of urban drainage and display differences (some subtle, others drastic) in scope and concept [36,37]. The terms are analyzed and their main characteristics and the scope of application of each of them are shown below.

### 2.1. Sustainable Drainage Systems (SuDS)

A term often used in the scientific literature is “Sustainable Drainage Systems (SuDS)”. Originally, the term SuDS described the British approach to sustainable urban drainage systems. During the 1990s, these systems developed specially in Scotland and Wales, with a strong regulatory push from the Scottish Environmental Protection Agency for implementation in new developments. Today, this term is mainly used in Europe, and it refers to a set of water management practices that aim to align modern drainage systems with natural water processes and are part of a broader green infrastructure strategy [38]. SuDS make urban drainage systems more compatible with components of the natural water cycle, such as storm overflows, soil percolation, and biofiltration, mitigating the effect that human development has had or may have on the natural water cycle, particularly surface runoff and water pollution trends [39].

These systems can be conventional infrastructure to reduce urban runoff (storm tanks), or vegetated areas also used to protect the principles and functions of natural ecosystems and provide a wide variety of benefits to people and wildlife [40]. SuDS are a complement to conventional sewer system infrastructure to minimize the hydrological impacts of urbanization and increase resilience to climate change in urban areas [41]. These measures are intended to limit extreme precipitation events [40] and are known to provide many environmental benefits [42], including the mitigation of climate change impact [43,44,45,46], along with ecological and social benefits and other potential long-term economically quantifiable benefits [47,48,49]. Types of SuDS include green roofs, permeable surfaces, wetlands, detention and infiltration basins, and filter drains, among others [50]. In general, these systems are used to support the transition to more sustainable and resilient environments [51,52] and their implementation is gradually progressing [53,54].

### 2.2. Low Impact Developments (LIDs)

Another widely used term is “Low Impact Developments (LIDs)”. LIDs were first conceptualized in the early 1990s by the Prince George’s County Department of Environmental Resources in the United States [55,56]. However, the term was first used by Burrill and Nolfi [57] in their study on reducing stormwater management costs. A manual on LID was then developed by Prince George’s County to increase its adaptability worldwide [58]. Nowadays this term is most commonly used in North America, Asia, and New Zealand and it is very similar to SuDS in origin, although has been used to characterize smaller-scale stormwater treatment devices. They are based on the regulation of stormwater at the source, through the use of control systems distributed at a micro-scale, such as the use of depression storage [55,59,60]. Today, optimal LID design is key in stormwater management, where the overall goal is to achieve a specific objective with limited available resources. The optimization objective can take many forms, such as reducing runoff volume, peak flow, combined sewer overflow volume, pollutant load, first flush volume, or minimizing cost. The optimal design of LIDs, such as the selection of appropriate LID, spatial layout, and size, can be obtained by considering an individual LID or a range of LIDs, under different storm scenarios or probabilistic rainfall events [55].

### 2.3. Best Management Practices (BMPs)

In North America and Canada, the most used term is “Best Management Practices (BMPs)”. Historically, the term has referred to auxiliary pollution controls in the fields of industrial wastewater control and municipal wastewater control [61], while in stormwater management (both urban and rural) and wetland management, BMPs can also refer to a primary control or treatment technique [62]. In fact, the US Environmental Protection Agency requires BMPs to satisfy wastewater permit applications with the advent of national pollution discharge elimination systems [35]. This term was coined in the 1990s as a way to describe acceptable practices that could be applied to protect water quality and promote soil conservation. They are methods that have been determined to be the most effective and practical means of preventing or reducing nonpoint source pollution to help achieve water quality objectives [61]. BMPs include both pollution prevention and mitigation measures [62]. Conservation buffers, including grassed watercourses, wetlands, and riparian areas, act as an additional protective barrier by capturing potential pollutants before they pass into surface waters [63,64]. Stormwater management in developed urban areas also uses BMPs to remove pollutants from runoff. BMPs include retention ponds, alum treatment systems, constructed wetlands, sand filters, baffle boxes, inlet devices, vegetated swales, buffer strips, and infiltration/exfiltration trenches. Storm drain signage programs are an educational BMP tool to remind people of the illegality of dumping trash, oil, pesticides, and other toxic substances into urban runoff drainage systems [65,66].

### 2.4. Water Sensitive Urban Design (WSUD)

In Australia, the most commonly used term is “Water Sensitive Urban Design (WSUD)” [67]. This phrase began to be used in the 1990s in Australia, with the first known reference to it being in 1992, although it did not come into widespread use until the beginning of the 21st century. In fact, Australia defined WSUD concepts in the 2004 National Water Initiative (NWI) as “Innovation and capacity building to create water-sensitive Australian cities” [68,69]. To apply this model in Australian cities, the Australian Government established a Cooperative Research Centre for Water Sensitive Cities (CRCWSC) in 2012 [69]. WSUD is a spatial planning and engineering design approach that integrates the urban water cycle, including stormwater, groundwater, and wastewater management and water supply, into urban design to minimize environmental degradation and enhance aesthetic and recreational appeal [70]. WSUD is described by Lloyd et al. [71] as an approach to urban planning and design that integrates with the urban water cycle aiming to minimize the hydrological impact of the urban development on its surroundings. It is practiced through both structural (green infrastructure systems, e.g., vegetated gardens, wetlands) and non-structural measures (i.e., policies aimed at improving water use efficiency) [72,73,74]. WSUD is associated with the consideration of multiple objectives that have traditionally been addressed separately: water security, public health, flood protection, waterway health, amenity, economic vitality, equity, and long-term sustainability [75,76,77,78,79,80]. Lloyd et al. [81] outline two fundamental aspects of WSUD: best management practices and best planning practices. While the former refers to structural and non-structural measures, the latter refers to the urban planning aspects of implementing distributed green systems. The WSUD concept integrates different stages of the urban water cycle into the urban design, such as water supply, stormwater, groundwater, and wastewater management [82]. This urban water model generates multiple benefits, including water supply, water quality, amenity, biodiversity, and urban heat mitigation [83].

### 2.5. Green Infrastructure (GI)

“Green Infrastructure (GI)” is a general term used to refer to the provision and maintenance of natural and semi-natural green spaces within the built “gray” infrastructure [84]. This term emerged in the USA in the 1990s and it seems to have origins in both landscape architecture, where it has been promoted as a network of green spaces, and in landscape ecology [35]. Today, this expression is used in all countries, especially in America, Europe, and Asia. It includes green spaces located, for example, in urban squares, pocket parks, sports fields, and cemeteries [85]. The difficulty of integrating green spaces into urban areas, which are almost fully occupied, has led to the implementation of hybrid systems that integrate greenery into or on top of gray infrastructure, such as green walls and roofs, permeable paving and roadside channels or gutters, shorelines, designated green belts, and walking paths in larger urban areas [86]. The benefits of integrating these practices in urban areas are very important for people’s health [87,88], air quality regulation, local temperature regulation, pollution abatement, and leisure opportunities [89,90,91]. Numerous recent studies claim that green infrastructures are the most effective practices to promote cooling through shading, airflow orientation, precipitation interception, and evapotranspiration [92,93,94,95,96,97]. The combination of green infrastructure with blue infrastructure (water bodies) can cool the overlying and adjacent air through evaporation and convection [98,99,100,101]. As has recently been proven in China, PM2.5 pollution concentration decreases as environmental greenness increases, a phenomenon that was observed across different land cover types and cities [102].

### 2.6. Nature-Based Solutions (NbS)

The last term commonly used to refer to practices for the mitigation of the effects of soil sealing is “Nature-based Solutions (NbS)”. This phrase was introduced towards the end of the 2000s by the World Bank to highlight the importance of biodiversity conservation for climate change mitigation and adaptation [103]. The term is used in all countries, especially in Europe. It is the most recent term, having been defined (in 2015) by the European Commission as “living solutions inspired by, continuously supported by, and using nature, which are designed to address various societal challenges in a resource-efficient and adaptive manner and to simultaneously provide economic, social and environmental benefits” [103]. The definition of NbS has recently been revised (in 2020) in the framework of the European Cooperation Action on Science and Technology Circular City as “concepts that bring nature into cities and those that derive from nature. NbS address societal challenges and enable resource recovery, climate mitigation and adaptation, human well-being, ecosystem restoration and/or improvement of biodiversity status, within urban ecosystems” [104]. However, the academic literature usually evaluates this term as a single functional dimension, usually water management [105]. NbS will play an important role in the EU Green Deal Strategy [106] and in the EU Biodiversity Strategy 2030 [107], aiming to implement the farm-to-table strategy [108]. They are also used in circular economy strategies and in the sustainability challenges of cities in the use of resources, such as water, energy and food [109]. In this sense they are now considered as a “critical element in addressing Sustainable Development Goals 11”, which is related to “sustainable cities and communities” [110], such as climate change adaptation measures [111], considering the cooling and urban heat mitigation effects of even small pocket green spaces in high density urban areas [112], or the specific benefits of vegetated green roofs and city trees on stormwater management for urban resilience [113].

By way of summary, the following table shows the main characteristics of the typologies of urban practices most commonly used to mitigate soil sealing in urban and peri-urban areas.

As can be seen in Table 1, the terms SuDS and LIDs are very similar in their definition and objectives. Both are based on water management to reduce the impact of extreme precipitation and re-naturalize the water cycle in urban areas. They are used to mitigate soil sealing in recently developed areas [114] and comprise different techniques, such as deep aquifer recharge through deep injection wells [115], as has been previously indicated. However, it is also relevant to consider their long-term performance with regard the evolution of their efficiency and clogging, effect in order to manage and maintain them adequately [116].

On the other hand, BMPs focus more on protecting water quality and promoting soil conservation in urban and peri-urban areas by reducing pollution. As for the terms GI and NbS, the analysis shows that they are also very similar and are based on the integration of green spaces into the urban environment, to improve the environmental quality of the surroundings, although in some cases GI may be linked to more specific techniques while NbS may have a broader scope. The term WSUD is an urban planning model that integrates the urban water cycle into the design of cities, minimizing the hydrological impact of urban development, and improving resilience to climate change.

Some recent tools have been developed in the assessment of SuDS and GI measures by determining surface runoff rates and helping in the reduction of flood hazards in critical zones [117]. Finally, another challenge for these practices will be the social acceptance of recycled stormwater use for non-potable residential purposes, where altruism and social and cultural norms will have significantly positive impact [118].

## 3. The Evolution of the Terminology Used for Urban Practices to Mitigate Soil Sealing

In order to obtain more information on the prevalence of soil sealing mitigation practices in recent years, an analysis was carried out on the articles published in the last 10 years (2012–2021). For this purpose, the terms already described above that represent the systems that are being used to mitigate the effects of soil sealing in urban and peri-urban areas have been taken as a basis.

The methodology used was based on that presented in the study by Fletcher et al. [35]. Their study analyzes the articles quoted in Google Scholar from 1980 to 2012, with keywords related to urban drainage and the study variable was the number of citations of the selected publications. In our study, however, data were extracted from the JCR database as it is considered of greater scientific relevance. The study period was from 2012 to 2021, and all recent urban practices for the mitigation of soil sealing were taken as keywords to expand the scope of study with respect to Fletcher’s article and the total number of publications was taken as the analysis variable, as it was considered more representative of the current scientific reality.

As mentioned in the Introduction, the keywords considered were the most used in 2012, at the beginning of the study period. Thus, the analysis was carried out with the following keywords: SuDS (Sustainable Drainage Systems), LIDs (Low Impact Developments), BMPs (Best Management Practices), WSUD (Water Sensitive Urban Design), GI (Green Infrastructures), and NbS (Nature-based Solutions). The search was conducted on the “Science Direct” website, which provides access to a large bibliographic database of scientific publications, one of the most important in the world. It hosts more than 18 million pieces of content from more than 4000 academic journals and 30,000 e-books. Only peer-reviewed articles written in English were considered. The search has quantified the number of articles published in the last 10 years that used selected terminology in the title, abstract, or keywords. The results obtained from this analysis were as follows.

**Figure 1:**
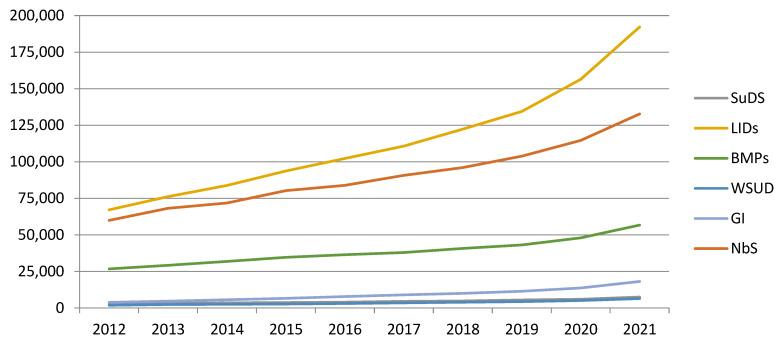
Evolution of scientific articles published on the topic of “soil sealing” according to the keywords analyzed.

Table 2 and Figure 1 show that the number of scientific articles published on the topic of “soil sealing” over the last 10 years has increased almost linearly, with a greater increase observed from 2019 onward, indicating that interest in this topic is increasing. In fact, the number of articles has doubled in the study period, from less than 200,000 to more than 400,000. This demonstrates the importance of this topic in the scientific field. In total, more than 2.5 million articles have been published on this topic in the last 10 years, indicating the great appeal of this topic for journals.

Figure 1 shows that by far the most frequently used term in scientific publications is LIDs, 26.3% more than the second most used term, NbS. The third most common term is BMPs, used in 33.8% of the LID value and 42.7% of the NbS value. SuDS, WSUD, and GI are the least used terms, far behind the first three. SuDS is used in 4% of the LID value, WSUD in 3%, and GI in 8%.

These results indicate that the terms used in America (LIDs and BMPs) are the most prevalent in the scientific literature, possibly because the number of scientific journals and scientists is greater in the US than in other countries. NbS appears in second place, despite being the most recently defined term. This indicates the importance this term has been gaining in recent years and that it may approach the usage of the terms LIDs and BMPs in the coming years. In addition, NbS has clearly exceeded that of GI, which is very similar in definition and objective/environmental benefit. Finally, we can see that the usage of all the above terms shows an increasing trend, indicating that the problem of soil sealing is becoming more and more important around the world.

## 4. Conclusions

This article has analyzed the main urban practices currently used to mitigate the effects of soil sealing in urban and peri-urban areas and their environmental benefits. It has been demonstrated that different terms are used to designate these practices, each with their own particularities but also with many common aspects. The terms most commonly used in the literature are SuDS (Sustainable Drainage Systems), LIDs (Low Impact Developments), BMPs (Best Management Practices), WSUD (Water Sensitive Urban Design), GI (Green Infrastructures), and NbS (Nature-based Solutions). It has been found that confusion can occur, with different authors using different terms to mean the same thing or ascribing different meanings to a given term. For instance, the terms SuDS and LID are very similar in their definition and objectives. They are based on water management to reduce the impact of extreme rainfall events and re-naturalize the water cycle in urban areas. However, BMPs focus more on protecting water quality and promoting soil conservation in urban and peri-urban areas by reducing pollution. GI and NbS, are more general terms and are based on the integration of green spaces in the urban environment, to improve the environmental quality of the surroundings. On the other hand, WSUD is an urban term that integrates the urban water cycle into the design of cities to minimize the hydrological impact of urban development and improve resilience to climate change. All these systems also share similarities in their environmental benefits for urban areas. Furthermore, it can be said that the new approaches have become increasingly sustainable, being more polyvalent and thus reflecting not only technical advances but also the relationship with nature and the cultural and social context.

An analysis of the presence of urban practices for soil sealing mitigation in the scientific literature over the last 10 years has been carried out using a selection on papers from the JCR database. Only peer-reviewed articles written in English in the JCR database were considered, which was the main limitation of the work, since non-English terminology used in other fields was not considered.

The evolution of studied publications shows that there was a linear growth in the use of this terminology, with a tendency to be exponential in recent years, which demonstrates that these practices are becoming increasingly relevant in the scientific field. The most commonly used terms are LIDs, NbS, and BMPs. LIDs and BMPs are terms used in North America, indicating that the number of articles published is higher in this area, probably due to the greater number of journals and scientists. However, NbS, despite being the most recently defined term, is in second place, showing that it is the fastest emerging term and could become the most prevalent in the coming years. On the other hand, the number of scientific articles published has increased over the last 10 years, doubling in this period. It can be deduced from the large quantity of articles published (2.5 million) that this topic is of increasing relevance and interest for the scientific journals and their readers. Moreover, the increasing usage of all terms analyzed suggests that the problem of soil sealing is an ever more imperative global issue.

In relation to the different terminology, this article shows that the meaning of the terms is different depending on the country and the moment and depending on the interpretation and the different conditions of each area. This variety and evolution in the terminology analyzed, as well as its definitions and objectives, makes it more difficult to homogenize actions, so this review helps to promote the necessity for a common terminology across countries in relation to soil sealing mitigation measures.

## Figures and Tables

**Table 1:** Urban practices to mitigate soil sealing; terms, definitions, and objectives [13,15,16,35,38,41,43,44,61,66,67,68,104].

Term	Areas Where It Is Most Used	Definition	Objectives/Environmental Benefits
SuDS	Europe	Water management practices to align conventional drainage systems with natural water processes	Absorb extreme rainfall and minimize the hydrological impacts of urbanization
LIDs	America, New Zealand and Asia	Stormwater management and control measures that are more sustainable than conventional approaches	Reduce runoff volume, peak flow, pollutant load, and first flush volume while minimizing cost
BMPs	America and Canada	Practices to protect water quality and promote soil conservation	Prevent or reduce nonpoint source pollution to help achieve water quality objectives
WSUD	Australia	Urban planning practices to integrate the urban water cycle in cities and minimize the hydrological impact of urban development	Integrate the urban water cycle (water supply, stormwater, groundwater, and wastewater) into urban design
GI	America, Europe and Asia	Provision and maintenance of natural and semi-natural green spaces within built “gray” infrastructure	Improve air quality regulation, regulate local temperature, decrease pollution, and create recreational opportunities
NbS	Europe	Solutions inspired and supported by nature which are cost-effective, to provide environmental, social and economic benefits.	Promote more ecological diversity and nature into cities and landscapes through locally adapted, resource-efficient, and systemic interventions.

**Table 2:** Number of scientific articles published on the topic of “soil sealing” according to the keywords analyzed.

	Number of Articles Published										
Keyword	2012	2013	2014	2015	2016	2017	2018	2019	2020	2021	Total
SuDS	2873	3150	3492	3696	3981	4505	4754	5430	5964	7451	45,296
LIDs	67,115	76,153	83,803	93,720	102,224	110,700	122,330	134,430	156,372	192,212	1,139,059
BMPs	26,697	29,125	31,830	34,595	36,370	37,886	40,724	43,110	47,967	56,676	384,980
WSUD	1808	2316	2487	2692	3032	3465	3891	4328	5070	6395	35,484
GI	3823	4621	5578	6548	7795	8921	9965	11,410	13,638	18,089	90,388
NbS	59,893	68,149	71,773	80,235	83,870	90,673	96,054	103,824	114,592	132,700	901,763
Total	162,209	183,514	198,963	221,486	237,272	256,150	277,718	302,532	343,603	413,523	2,596,970

## Data Availability

Data-supporting reported results can be found in https://www.sciencedirect.com/, accessed on 17 January 2022.
